# A case of interstitial granulomatous dermatitis presenting in a patient with necrotizing sarcoid granulomatosis

**DOI:** 10.1016/j.jdcr.2023.06.001

**Published:** 2023-06-14

**Authors:** Rachel Elsanadi, Katerina Yale, Nathan Rojek, Dani Zhao, Bonnie Lee, Michelle S. Min

**Affiliations:** a Department of Dermatology, University of California, Irvine, Irvine, California; b Department of Dermatopathology, University of California, Irvine, Irvine, California

**Keywords:** autoimmune disease, dermatologic rheumatology, dermatopathology, diagnosis of exclusion, hydroxychloroquine, inflammation, inflammatory, interstitial granulomatous dermatitis, necrotizing sarcoid granulomatosis, rash, reactive, prednisone

## Introduction

Interstitial granulomatous dermatitis (IGD) is a rare reactive granulomatous dermatitis which typically presents in the setting of underlying inflammation.^1^^,^^2^ Numerous associations have been described including connective tissue disease, malignancy, infection, medications, and other systemic disorders, such as sarcoidosis.^1^, ^2^, ^3^, ^4^, ^5^, ^6^ However, to our knowledge, IGD has not yet been reported in association with necrotizing sarcoid granulomatosis (NSG), which is a rare pulmonary disease with necrosis and vasculitis.^7^^,^^8^ Herein, we present and discuss a case in which these conditions appeared together.

## Case report

**Fig 1:**
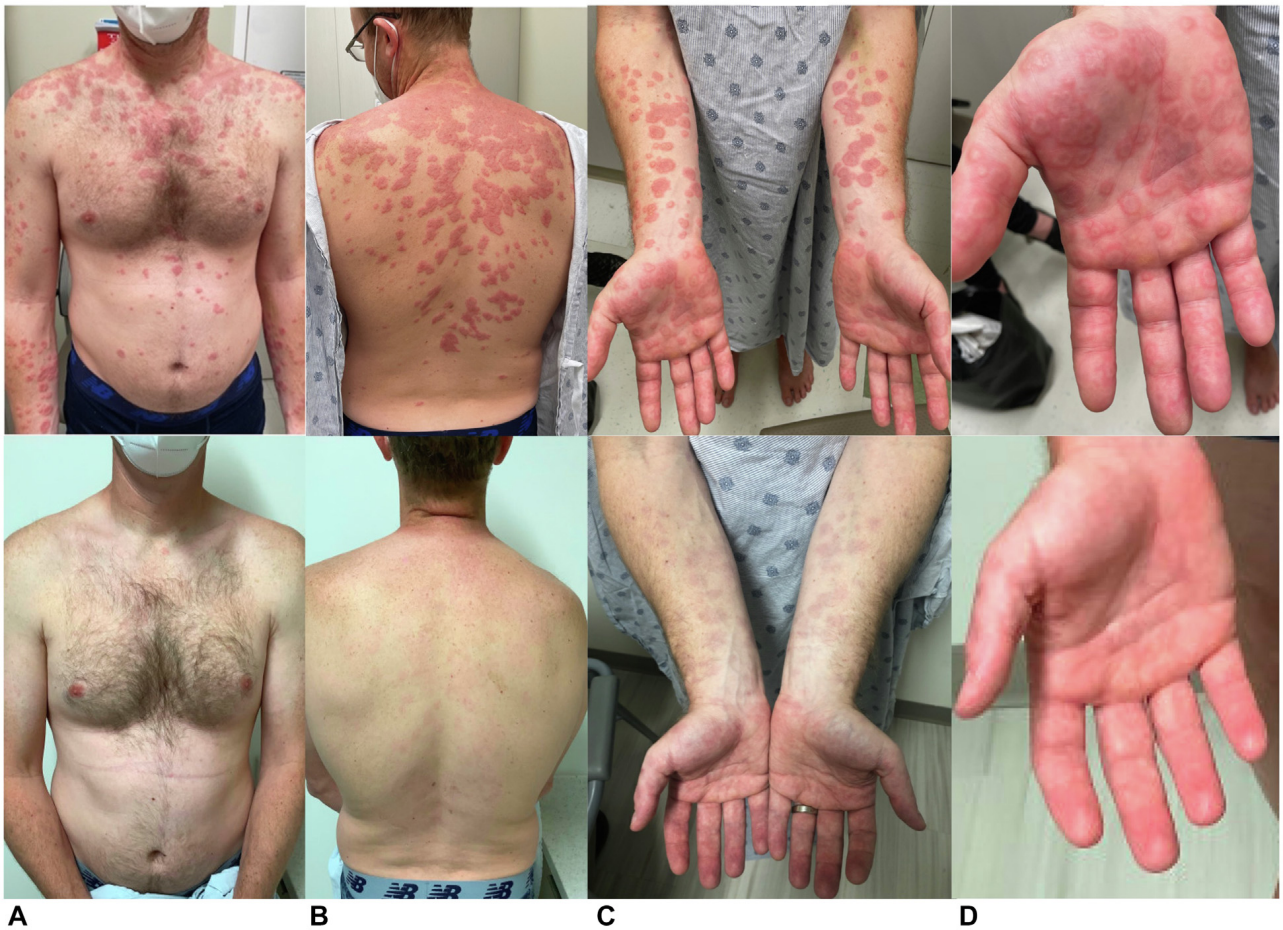
Clinical images of interstitial granulomatous dermatitis of the (**A**) chest, (**B**) back, (**C**) upper extremities, and (**D**) palms before and 4 months after treatment initiation, in a male diagnosed with necrotizing sarcoid granulomatosis.

A previously healthy 36-year-old male presented with a 2-week, rapidly progressive rash, and painful hand swelling preceded by fever, myalgias, fatigue, and night sweats. He denied respiratory symptoms. He denied new medications or supplements. Family history was significant for a father with breast cancer and a maternal cousin with lupus erythematosus. Examination revealed numerous, coalescing, erythematous-to-violaceous annular plaques with indurated borders on the scalp, forehead, trunk, and extremities including palms (Fig 1, *A*-*D*).

**Fig 2:**
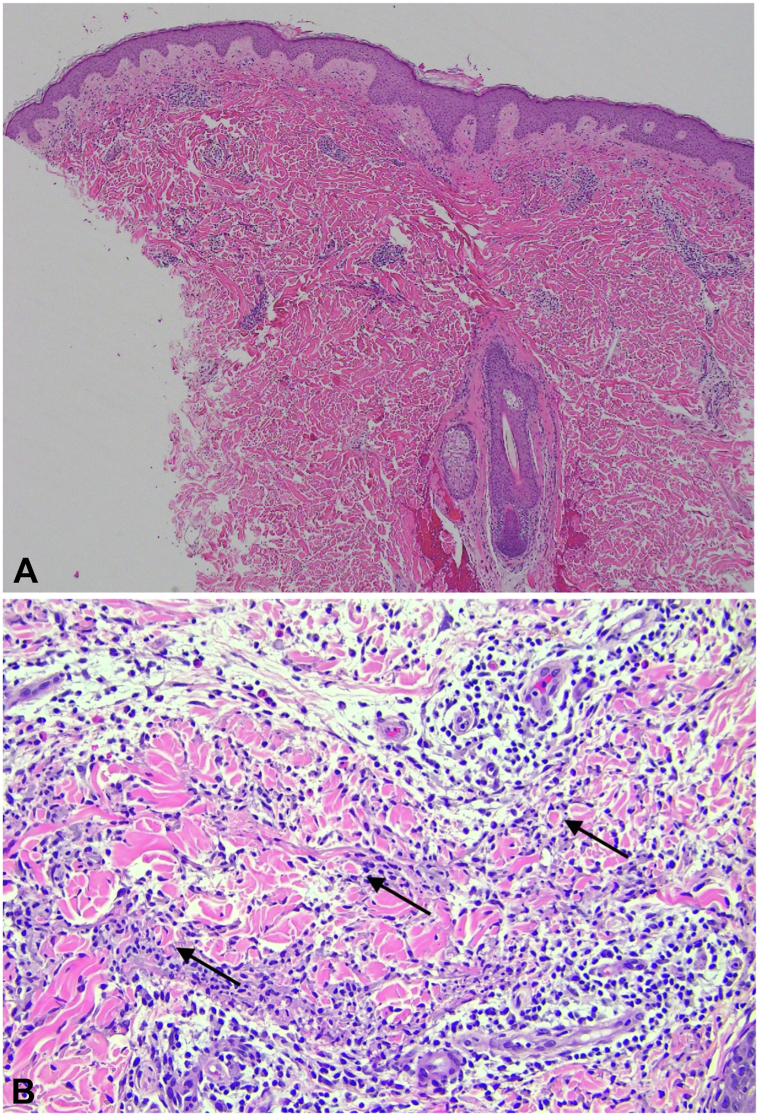
Histologic images of interstitial granulomatous dermatitis: H&E at (**A**) 4× magnification shows a superficial perivascular and interstitial inflammatory cell infiltrate, and at (**B**) 20×, a histiocytic infiltrate between collagen bundles with an *arrow* indicating the “floating sign.”

Skin biopsy revealed superficial perivascular inflammation with lymphocytes, eosinophils, and plasma cells, as well as small aggregates of histiocytes between collagen bundles. Colloidal iron stain failed to highlight increased mucin. Special stains (acid-fast bacilli, Fite, and Grocott’s methenamine silver) failed to demonstrate evidence of infection. These findings favored a diagnosis of IGD (Fig 2, *A* and *B*).

A complete blood count, comprehensive metabolic panel, and urinalysis were within normal limits; however, erythrocyte sedimentation rate (42 millimeter/hour) and C-reactive protein (6.1 milligram/deciliter) were elevated. Free thyroxine was slightly elevated; however, thyroid stimulating hormone (TSH) was within normal limits. An extensive infectious and autoimmune workup (including but not limited to serologic fungal tests, interferon-gamma release assay for tuberculosis, anti-nuclear antibody, and rheumatoid factor) was performed with negative results.

Chest imaging, both x-ray and computed tomography (CT), revealed bilateral hilar lymphadenopathy. Right lobe bronchoscopy revealed necrotizing granulomas, raising the concern for infection. Bronchoalveolar lavage was negative for infection (sputum gram stain, acid-fast bacilli culture, fungal culture), and no malignant cells were seen. Therefore, a diagnosis of interstitial granulomatous dermatitis (IGD) in the setting of necrotizing sarcoid granulomatosis (NSG), a rare pulmonary disorder, was made.

The patient was started on high-dose oral prednisone and hydroxychloroquine for his rash, which rapidly responded to therapy, fully resolving within 3 months (Fig 1, *A*-*D*).

## Discussion

While the classic clinical morphology of IGD involves linear cords on the lateral trunk and proximal inner limbs known as the “rope sign”, this is clinically observed in only 10% of cases.^1^^,^^2^ Multiple asymptomatic skin-colored to erythematous papules or plaques dispersed symmetrically on the trunk and extremities is far more common.^1^^,^^2^ Depending on the presentation, IGD may mimic other disorders.^1^^,^^2^ In this case, there was initial clinical concern for erythema multiforme given the nearly targetoid plaques on the palms.

Because IGD is a reactive condition, investigation for an underlying etiology is critical. IGD is most associated with underlying autoimmune disease, specifically, rheumatoid arthritis.^1^^,^^2^^,^^9^ Other common associations include connective tissue disease, hematologic malignancy, and certain drugs.^1^ Typical histologic presentation involves a dense infiltrate of interstitial histiocytes surrounding areas of abnormal and degenerating collagen, also termed “the floating sign.” (Fig 2, *B*).^1^ Eosinophils and neutrophils are less common, and there is absence of vasculitis. Little to no mucin is present and infiltrate can extend deeper, differentiating IGD from granuloma annulare, which can otherwise share histologic similarities.^1^

In this case, the patient’s rash was associated with necrotizing sarcoid granulomatosis, an exceedingly rare noninfectious granulomatous pulmonary disease.^7^ It typically affects women more than men and primarily involves the lungs but may also involve the central nervous system, skin, or other organs.^7^ The classic triad entails sarcoid-like granulomas with variable levels of necrosis, granulomatous vasculitis, and pulmonary nodules.^7^^,^^8^ There may also be hilar or mediastinal lymphadenopathy on imaging.^10^ Cases may be asymptomatic or present with fever, nonproductive cough, dyspnea, or chest pain.^10^

Necrotizing sarcoid granulomatosis (NSG) is a diagnosis of exclusion.^10^ Therefore, differential diagnoses such as infection (particularly tuberculosis), granulomatosis with polyangiitis, or nodular sarcoidosis must also be ruled out.^10^ While it is controversial whether NSG represents a subset of sarcoidosis or a separate category of disease, NSG usually follows a benign clinical course and may be effectively treated with steroids.^7^^,^^8^^,^^10^

Though IGD has not been linked to NSG in the literature, there have been reports of both IGD and palisaded neutrophilic and granulomatous dermatitis (PNGD) in association with sarcoidosis.^1^^,^^5^^,^^6^ Of note, while IGD and PNGD may present with distinct features, there is considerable overlap between the disorders both clinically and histologically. Reactive granulomatous dermatitis (RGD) may serve as a unifying term.^1^^,^^9^ As in this case, clinicians should maintain a high level of suspicion for a variety of diseases that may be associated with reactive granulomatous dermatitis (RGD). The authors recommend chest imaging if other work-up returns negative.

Treatment of the underlying systemic disease will often result in resolution of IGD.^1^ Systemic corticosteroids and hydroxychloroquine, which were used in this case, have been reported as effective treatments, as well as dapsone, non-steroidal anti-inflammatory drugs, etanercept, and topical steroids.^1^ In our case, the patient was able to taper off high-dose prednisone (initiated at 0.85 mg/kg) over the course of 3 months and has not had recurrence of IGD with maintenance hydroxychloroquine (200 milligrams twice daily).

This case highlights a previously unreported association of IGD with NSG, which is a rare systemic disease. While NSG is exceedingly rare, this report undermines the necessity of extensive laboratory and imaging workup in patients who present with IGD, as IGD is considered to be a reactive condition.

## Conflicts of interest

Min has served on the advisory board for Horizon.
